# Genetic Polymorphisms in *ZFHX3* Are Associated with Atrial Fibrillation in a Chinese Han Population

**DOI:** 10.1371/journal.pone.0101318

**Published:** 2014-07-01

**Authors:** Yaowu Liu, Bixian Ni, Yuan Lin, Xin-guang Chen, Zhen Fang, Liyan Zhao, Zhibin Hu, Fengxiang Zhang

**Affiliations:** 1 Department of Cardiology, the First Affiliated Hospital of Nanjing Medical University, Nanjing, Jiangsu, China; 2 Department of Epidemiology and Biostatistics, School of Public Health, Nanjing Medical University, Nanjing, Jiangsu, China; Loyola University Chicago, United States of America

## Abstract

**Background:**

The gene zinc finger homeobox 3 (*ZFHX3*) encodes a transcription factor with cardiac expression and its genetic variants are associated with atrial fibrillation (AF). We aimed to explore the associations between single nucleotide polymorphisms (SNPs) of *ZFHX3* and the risk of AF in a Chinese Han population.

**Methods:**

We genotyped eight SNPs, including seven potentially functional SNPs and one previously reported SNP by using the middle-throughput iPLEX Sequenom MassARRAY platform. Odds ratios (ORs) and 95% confidence intervals (CIs) were calculated in logistic regression models.

**Results:**

We enrolled a total of 1,593 Chinese Han origin individuals in the study, including 597 AF patients and 996 non-AF controls. Logistic regression analyses revealed that potentially functional SNPs rs6499600 and rs16971436 were associated with a decreased risk of AF (adjusted OR  = 0.73, 95% CI: 0.63–0.86, *P* = 1.07×10^−4^; adjusted OR  = 0.74, 95% CI: 0.56–0.98, *P* = 0.039, respectively). In addition, rs2106261 showed a robust association with an increased risk of AF (adjusted OR  = 1.71, 95% CI: 1.46–2.00, *P* = 1.85×10^−11^). After multiple comparisons, rs16971436 conferred a borderline significant association with the risk of AF. Stratification analysis indicated that the risks of AF were statistically different among subgroups of age for rs2106261, and the effect for rs16971436 was more evident in subgroups of patients with coronary artery disease.

**Conclusion:**

In summary, our study investigated the role of genetic variants of *ZFHX3* in AF and two SNPs (rs2106261, rs6499600) showed significant associations while rs16971436 conferred a borderline significant association with AF risk in Chinese Han populations. However, further large and functional studies are warranted to confirm our findings.

## Introduction

Atrial fibrillation (AF) is the most prevalent sustained arrhythmia in clinical practice characterized by uncoordinated atrial activation. The prevalence of AF was estimated at approximately 2% of the general population [1], [2] and this arrhythmia accounts for nearly one-third of hospitalizations for cardiac rhythm disturbances [3]. It is closely related to a 5-fold risk of stroke, a 3-fold incidence of congestive heart failure, and higher all-cause mortality, which may contribute to high healthcare cost (average 2000 EUR per AF patient per year) [4]–[6].

AF frequently occurred coupled with multiple cardiovascular risk factors such as advancing age, male sex, hypertension, diabetes, ischemic, valvular heart disease, heart failure, and hyperthyroidism [7]–[9]. However, some patients maintain sinus rhythm regardless of various risk factors for AF while some other patients develop AF without any evidence of AF-related diseases [10]. Epidemiologic studies have shown that first-degree relatives of AF patients were 1.77-fold to 4.67-fold more likely to have AF than the general population, and offspring with one parent suffered from AF had approximately a 2-fold increase in the risk of developing AF [11], [12]. These facts indicate that predisposing genetic factors may affect AF susceptibility. Over the past years, lots of genetic studies of AF were performed by applying several techniques such as linkage analysis, candidate gene resequencing and association studies. Using these methods, especially genome wide association studies (GWASs), investigators have identified multiple genes and genetic loci which are associated with AF [13], [14]. In 2009, Benjamin et al. [15] conducted a meta-analysis of GWASs for AF and identified a locus for AF (*ZFHX3*, rs2106261, RR  = 1.19; *P* = 2.7×10^−7^). Candidate gene studies are approaches employed by researchers to screen variants in any gene that is hypothesized to cause a disease. We sought to identify potentially functional AF susceptibility SNPs of *ZFHX3* using the candidate gene tool. Furthermore, we aim to confirm the association of rs2106261 in *ZFHX3* with AF risk in Chinese Han populations.

## Materials and Methods

### Study population

This case-control study was approved by the Ethical Committee Review Board of Nanjing Medical University, China. All the participants enrolled in the study gave their written informed consent.

In brief, the cases were incident AF patients recruited from Department of Cardiology, the First Affiliated Hospital of Nanjing Medical University from June 2010 to August 2013. All AF cases in the study were confirmed according to the diagnostic criteria, 2011 ACCF/AHA/HRS focused updates incorporated into the ACC/AHA/ESC 2006 guidelines for the management of patients with atrial fibrillation [3]. Physical examinations were carried out by expert cardiologists using routine 12-lead electrocardiography (ECG), and/or ambulatory ECG recordings. According to clinical characteristics, AF can be classified into paroxysmal AF (episodes that generally last 7 days or less), persistent AF (episodes that sustain beyond 7 days) and permanent AF (ongoing long-term episodes, in which cardioversion has failed or has not been attempted). Young AF individuals (under 60 years) without clinical or echocardiographic evidence of cardiopulmonary disease, including hypertension were considered as lone AF [16]. The control subjects were genetically unrelated inpatient individuals from multiple departments of the First Affiliated Hospital of Nanjing Medical University. All the controls were confirmed to be free of AF based on ECG or medical files at the time of enrollment.

We also collected general and clinical information from medical recording files in the hospital system such as age, gender, and history of hypertension, diabetes, coronary artery disease (CAD), and hyperthyroidism. Patients with hyperthyroidism, severe cardiac dysfunction (NYHA Class IV) and advanced age (beyond 90 years) were excluded in both AF and control groups. All the enrolled individuals are of the ethnic Chinese Han origin by self-report.

### SNP selection

We first used public HapMap SNP database (phase II + III Feb 09, on NCBI B36 assembly, dbSNP b126) to search SNPs that localized within the gene region of *ZFHX3* (including 10 kb up-stream region of the gene), with MAF≥0.05 in Chinese Han population (CHB). Then, a web-based analysis tool was used to predict the function of these SNPs (http://snpinfo.niehs.nih.gov/snpinfo/snpfunc.htm). After function prediction analysis, a total of 9 potentially functional SNPs were selected. Using the Haploview 4.2 software, linkage disequilibrium (LD) analysis with an r^2^≥0.8 was further applied to filter these functional SNPs and 7 (rs12596992, rs13336412, rs16971312, rs16971436, rs17680796, rs6499600, rs8049936) of which were remained. Additionally, previously reported single nucleotide polymorphism (SNP) rs2106261 in *ZFHX3* was selected as well for this study. As a result, 8 loci were finally determined to perform genotyping.

### SNP genotyping

Blood samples were drawn from study participants and genomic DNA was isolated from EDTA-preserved whole blood, using the standard phenol-chloroform method [17]. The genotyping was performed by using the middle-throughput iPLEX Sequenom MassARRAY platform (Sequenom, Inc, San Diego, CA, USA). All SNPs were successfully genotyped with call rates >95%.

### Statistical analysis

The standard independent samples *t*-test was used to compare continuous variables and the χ^2^ test was used to compare categorical variables. The Hardy-Weinberg equilibrium comparing the observed genotype frequencies with the expected ones was determined by the χ^2^ test in the control group. Odds ratios (ORs) and 95% confidence intervals (CIs) were calculated using logistic regression analyses in the additive model to assess the strength of the associations between the variants genotypes and AF risk. The heterogeneity of associations between subgroups was assessed using the χ^2^-based Q-test. All statistical analyses were performed using STATA 12.0 software (Stata Corporation, College Station, TX, USA) and *P*<0.05 was considered significant.

## Results

### Characteristics of the Study Population

A total of 1,593 Chinese Han origin individuals were recruited in this study, including 597 AF patients and 996 non-AF controls. The demographic characteristics of cases and controls are summarized in **Table 1**. As shown, there were no significant differences for the distributions of age and gender between cases and controls (*P* values were 0.278 and 0.630, respectively). The mean age of AF and control groups was 58.4±11.5 and 59.0±10.2 years, respectively. Male proportion was 66.5% and 67.7% in AF group and control group, separately. Among cases, 64.2% patients were paroxysmal AF, 32.8% were persistent AF, while others were permanent AF. And patients with lone AF accounted for a small part (11.9%) of all AF cases. Considered as common risk factors for AF, hypertension, diabetes, and CAD were more prevalent in cases than controls (*P*<0.05).

**Table 1:** Clinical characteristics in AF cases and AF-free controls.

Variants	Cases (N = 597)	Controls (N = 996)	*P* value
Male gender (%)	397(66.5%)	674(67.7%)	0.630
Age, years	58.4±11.5	59.0±10.2	0.278
Paroxysmal AF (%)	383 (64.2%)	NA	-
Persistent AF (%)	196 (32.8%)	NA	-
Permanent AF (%)	18 (3.0%)	NA	-
Lone AF (%)	71 (11.9%)	NA	-
Hypertension (%)	260 (43.6%)	267 (26.8%)	<0.001
Diabetes (%)	53 (8.9%)	28 (2.8%)	<0.001
CAD (%)	48 (8.0%)	51 (5.1%)	0.019

Data are presented as mean ± standard deviation or number (percentage).

AF, atrial fibrillation; CAD, coronary artery disease; NA, not available.

### Overall Associations between *ZFHX3* Variants and AF risk

The genotype distributions of the 8 polymorphisms and their associations with AF were shown in **Table 2**. The observed genotype frequencies for these SNPs were all in agreement with Hardy–Weinberg in the controls. Logistic regression analyses revealed that functional SNPs rs6499600 and rs16971436 were associated with decreased risks of AF in the additive model (adjusted OR = 0.73, 95% CI: 0.63–0.86, *P* = 1.07×10^−4^; adjusted OR  = 0.74, 95% CI: 0.56–0.98, *P* = 0.039, respectively). In addition, rs2106261 showed a robust association with an increased risk of AF (adjusted OR  = 1.71, 95% CI: 1.46–2.00, *P* = 1.85×10^−11^). We further calculated *P* values for false discovery rate (*P*-FDR) to perform multiple comparisons. After comparisons, we found that rs6499600 and rs2106261 remained their associations with AF risk while rs16971436 lost its association with the risk of AF. There were no obvious evidences of significant association between other 5 SNPs and AF risk.

**Table 2:** Summary of associations between 8 SNPs in *ZFHX3* and the risk of AF.

SNP	Position	Minor/major allele	Cases^a^	Controls^a^	MAF(cases)	MAF(controls)	*P*_HWE_	OR(95%CI) b	*P* b	*P*_FDR_ c
rs12596992	71536544	C/G	96/252/236	132/458/382	0.38	0.37	0.78	1.08(0.93–1.26)	0.316	0.316
rs13336412	71539450	A/C	124/297/173	195/500/301	0.46	0.45	0.62	1.1(0.94–1.27)	0.23	0.263
rs16971312	71413345	G/A	43/226/326	78/426/492	0.26	0.29	0.28	0.85(0.72–1.00)	0.056	0.112
rs16971436	71550264	G/T	3/71/523	11/152/833	0.06	0.09	0.18	**0.74(0.56–0.98)**	**0.039**	0.104
rs17680796	71538441	T/C	60/266/268	84/438/472	0.32	0.3	0.21	1.15(0.98–1.35)	0.09	0.144
rs6499600	71536875	T/C	75/275/244	164/506/317	0.36	0.42	0.11	**0.73(0.63–0.86)**	**1.07×10^−4^**	**4.28×10^−4^**
rs2106261	71609121	A/G	110/299/184	99/446/451	0.44	0.32	0.46	**1.71(1.46–2.00)**	**1.85×10^−11^**	**1.48×10^−10^**
rs8049936	71642787	G/A	61/246/284	114/435/447	0.31	0.33	0.6	0.9(0.77–1.05)	0.175	0.233

SNP, single nucleotide polymorphism; AF, atrial fibrillation; MAF, minor allele frequency; *P*_HWE_, *P* values for Hardy–Weinberg equilibrium tests in control groups; OR odds ratio; CI confidence interval.

a Individuals homozygous for the minor allele/heterozygous/homozygous for the major allele.

b OR (95%CI) and *P* values were derived from logistic regression analysis in the additive model adjusting for age, gender, hypertension, diabetes and coronary artery disease.

c Multiple comparisons *P* values for false discovery rate.

### Stratification Analysis

In the stratification analysis, we further evaluated the associations of rs6499600, rs16971436 and rs2106261 on AF risk in subgroups based on age, gender, hypertension, diabetes and CAD. As shown in **Table 3**, a significant difference among subgroups of age was observed for the association of rs2106261 with AF risk (*P* for heterogeneity  = 0.001). Patients with CAD exhibited more significant protective effect than patients who were free of CAD for rs16971436 (adjusted OR  = 0.13, 95% CI: 0.02–0.71).

**Table 3:** Stratified analysis for associations of rs6499600, rs16971436 and rs2106261 in *ZFHX3* with AF risk.

Variables	Cases	Controls	Adjusted OR(95%CI)^a^	*P* b	Cases	Controls	Adjusted OR(95%CI)^a^	*P* b	Cases	Controls	Adjusted OR(95%CI)^a^	*P* b
SNP	rs6499600(TT/TC/CC)				rs16971436(GG/GT/TT)				rs2106261(AA/AG/GG)			
Age^c^												
≤51	19/74/74	51/117/60	0.54(0.39–0.74)	0.099	0/22/146	1/34/196	0.83(0.46–1.50)	0.618	43/87/38	22/86/123	2.56(1.87–3.52)	0.001
52∼60	20/74/63	48/151/92	0.75(0.55–1.01)		1/17/139	5/53/236	0.55(0.32–0.95)		37/68/52	26/126/142	2.00(1.49–2.69)	
61∼66	16/65/48	35/111/83	0.93(0.67–1.29)		1/13/115	1/27/202	0.90(0.47–1.73)		12/72/44	27/122/81	1.00(0.70–1.42)	
≥67	20/62/59	30/127/82	0.81(0.58–1.13)		1/19/123	4/38/199	0.83(0.47–1.46)		18/72/50	24/112/105	1.49(0.99–1.98)	
Gender												
Males	55/178/161	100/352/216	0.79(0.65–0.96)	0.131	1/45/351	7/107/560	0.65(0.45–0.93)	0.299	79/199/116	64/294/316	1.86(1.53–2.25)	0.070
Females	20/97/83	64/154/101	0.61(0.46–0.81)		2/26/172	4/45/273	0.92(0.57–1.47)		31/100/68	35/152/135	1.37(1.04–1.82)	
Hypertension												
Yes	36/130/94	37/140/86	0.91(0.70–1.19)	0.066	0/32/228	2/42/223	0.68(0.42–1.10)	0.687	44/129/87	17/126/124	1.78(1.35–2.34)	0.716
No	39/145/150	127/366/231	0.65(0.53–0.79)		3/39/295	9/110/610	0.77(0.55–1.10)		66/170/97	82/320/327	1.67(1.37–2.02)	
Diabetes												
Yes	7/27/19	7/13/8	0.70(0.30–1.66)	0.91	0/6/47	2000-3-25	1.62(0.27–9.63)	0.713	6/23/22	1/14/13	1.48(0.56–3.91)	0.764
No	68/248/225	157/493/309	0.74(0.63–0.87)		3/65/476	11/149/808	0.74(0.56–0.99)		104/276/162	98/432/438	1.74(1.48–2.04)	
CAD												
Yes	3/27/17	10/26/15	0.81(0.39–1.69)	0.579	0/2/46	0/10/41	0.13(0.02–0.71)	0.001	7/23/17	2/26/23	1.33(0.64–2.76)	0.486
No	72/248/227	154/480/302	0.74(0.63–0.88)		3/69/477	11/142/792	0.81(0.61–1.08)		103/276/167	97/420/428	1.72(1.46–2.03)	

CAD, coronary artery disease.

a Obtained in logistic regression models with adjustment for age, gender, hypertension, diabetes and coronary artery disease (the stratified factor in each stratum excluded).

b *P* for heterogeneity test using the Chi-square-based Q test.

c Age was divided into four sub-groups according to its median (60 years), lower quartile (51 years) and upper quartile (66 years).

## Discussion

In this study, we investigated the associations between polymorphisms of *ZFHX3* and the risk of AF in a case-control study including 597 cases and 996 controls. Seven potentially functional SNPs and one previously reported polymorphism in ZFHX3 were selected for genotyping in our population. Two SNPs (rs6499600 and rs2106261) were identified to be significantly associated with AF risk and rs16971436 conferred a borderline significant association with the risk of AF in Chinese Han populations. Forrs2106261, the effects were statistically different among subgroups of age in stratified analysis, and the risk associated with rs16971436 was more evident in patients with CAD.

*ZFHX3* (also known as *ATBF1*), encodes a transcription factor with multiple homeodomains and zinc finger motifs, and regulates myogenic and neuronal differentiation. Little is known about the function of *ZFHX3* for cardiac expression, but it has been shown to interact with Protein Inhibitor of Activated Stat3 (PIAS3) [18]. STAT3 was found to be a regulator of paracrine circuits in the heart, and it was essential for interstitial matrix deposition balance and capillary vasculature maintenance [19]. Increased expression of STAT3 has been observed in animal models of AF and proposed to contribute to atrial matrix deposition. Tsai *et al*. suggested that activated angiotensin II/Rac1/STAT may be associated with or perhaps contribute to the structural and inflammatory changes in AF [20].

A total of 3 SNPs were significantly associated with the risk of AF (rs2106261, rs6499600 and rs16971436) in our population. The SNP rs2106261 locates in the first intron of *ZFHX3* and has been reported to confer increased risk of AF in European and Chinese populations [15], [21]. Even though the mechanism of rs2106261 conferred increased susceptibility of AF is little known, one plausible hypothesis is that it may alter the expression of *ZFHX3* by modulating regulation of *ZFHX3* transcription or pre-mRNA splicing because intronic sequences have been found to play regulatory roles in gene expression recently [22]. The SNP rs6499600 is located in the seventh intron of *ZFHX3*. And it may affect the binding of transcription factors such as ATF6 (activating transcription factor 6), which was predicted by the online tool SNPinfo (http://snpinfo.niehs.nih.gov/index.html). ATF6 is a key transcriptional activator to maintain cellular homeostasis, and inhibition of which induced dilatation of left ventricle and depression of cardiac function in murine heart [23]. *ZFHX3* rs16971436 is a T to G variant at the codon of 428 of exon 1, resulting in an amino acid alteration from threonine (Thy) to proline (Pro). Therefore, we proposed that the missense variant might lead to the change of *ZFHX3* expression, consequently cause a dysregulation of STAT3 and finally result in altered susceptibility of AF.

Several limitations of the present study need to be addressed. Firstly, we did not replicate the results in additional individuals, this may contribute to potential false positive errors. The present analysis was restricted to individuals of Chinese Han descent, and therefore, the findings may not be generalizable to individuals of other races and ethnicities. Secondly, as echocardiography results were unable to be obtained from every participant, left ventricular ejection fraction (LVEF) values were not measured to evaluate cardiac function in both groups. Also, we did not analyze the impact of SNPs on stroke because some patients enrolled in the study had not performed head CT or MRI scans. Furthermore, the way to select potentially functional SNPs as target ones by using web-based tool may bring some positive or negative errors. Therefore, the results are required to be further replicated by well-designed studies in additional large-scale Chinese Han populations.

In conclusion, our study investigated the role of genetic variants of *ZFHX3* in AF and two SNPs (rs2106261, rs6499600) showed significant associations while rs16971436 conferred a borderline significant association with AF risk in Chinese Han populations. However, further large and functional studies are warranted to confirm our findings.
